# Podocytic infolding in Schimke immuno-osseous dysplasia with novel SMARCAL1 mutations: a case report

**DOI:** 10.1186/s12882-020-01809-6

**Published:** 2020-05-11

**Authors:** Shiqiu Xiong, Lanjun Shuai, Xiaoyan Li, Xiqiang Dang, Xiaochuan Wu, Qingnan He

**Affiliations:** Department of Pediatrics, the Second Xiangya Hospital, Central South University, Changsha, 410011 Hunan China

**Keywords:** Schimke immuno-osseous dysplasia, Podocytic infolding glomerulopathy, Nephrotic syndrome

## Abstract

**Background:**

Schimke immuno-osseous dysplasia (SIOD) is a rare autosomal recessive disorder characterized by spondyloepiphyseal dysplasia, progressive renal insufficiency and defective cellular immunity. Podocytic infolding glomerulopathy (PIG) is a newly proposed disease entity characterized by microspheres or microtubular structures associated with podocytes infolding into the glomerular basement membrane (GBM) on electron microscopy (EM).

**Case presentation:**

A 4-year-old boy was admitted to our ward due to proteinuria and edema lasting 1 month. He had a short trunk and demonstrated subtle dysmorphology, with a triangular shape, a broad nasal bridge and a bulbous nasal tip. The laboratory findings were as follows: lymphocytes, 0.5 × 10^9^/L; urine protein, 3.67 g/d; albumin, 9.8 g/L; and cholesterol, 11.72 mmol/L. Skeletal X rays showed small iliac wings, small ossification centers of the capital femoral epiphyses, shallow dysplastic acetabular fossae and mildly flattened vertebrae. The specimen for light microscopy (LM) suggested focal segmental glomerulosclerosis (FSGS). EM revealed a focal thickness of the GBM with some cytoplasmic processes of podocyte infolding into the GBM. Gene sequencing showed novel compound heterozygous mutations in the SMARCAL1 gene (c.2141 + 5G > A; c.2528 + 1G > A) that were inherited from his parents. Finally, we established the diagnosis of SIOD and treated him with diuretics and angiotensin-converting enzyme inhibitors (ACEIs).

**Conclusion:**

The pathogenic mechanism of PIG has not been clarified. Further studies are required to understand whether gene mutations, especially those related to podocytes, contribute to the pathogenesis of podocytic infolding.

## Background

Schimke immuno-osseous dysplasia (SIOD) is an autosomal recessive inherited disease in which the SMARCAL1 gene is mutated on chromosome 2; SIOD is mainly characterized by spondyloepiphyseal dysplasia, lymphopenia with defective cellular immunity, and progressive renal dysfunction [1]. Hypothyroidism, bone marrow failure, and episodic cerebral ischemia have also been reported [2]. Patients with SIOD are resistant to various immunosuppressants. Histopathology of the kidney in most of the patients shows FSGS [2]. PIG is a rare and peculiar glomerulopathy in which the ultrastructural finding shows podocyte infolding and invagination into the GBMs, characterized by microspherules and microtubules on EM [3]. Only 31 cases have been reported worldwide to date, and almost two-thirds of the patients were diagnosed with connective tissue disease [4]. To date, no case of SIOD has been reported in which kidney histopathology indicates podocytic infolding.

## Case presentation

**Fig. 1:**
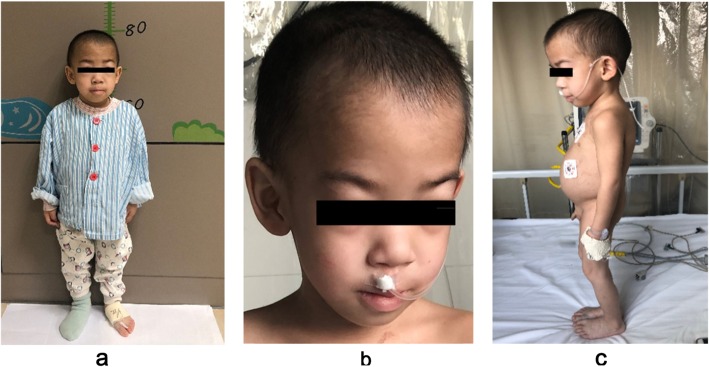
The patient had a short trunk with a height of 81 cm (**a**), and demonstrated subtle dysmorphology, with a triangular shape, a broad nasal bridge and a bulbous nasal tip (**b**). He had lumbar lordosis and a protruding abdomen (**c**)

**Fig. 2:**
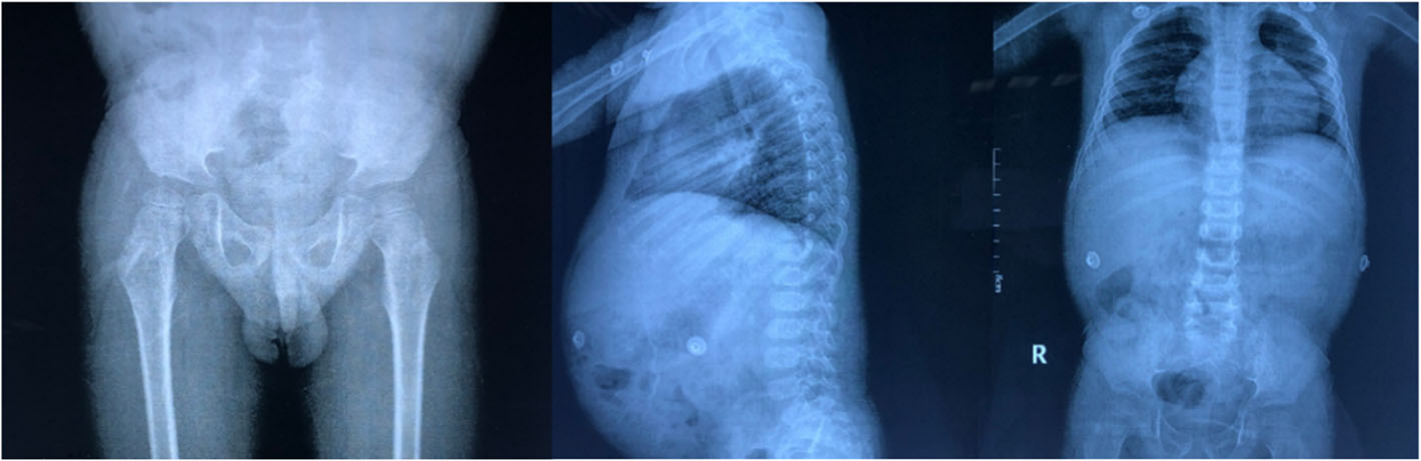
Skeletal X rays show small iliac wings, small ossification centers of the capital femoral epiphyses, shallow dysplastic acetabular fossae and mildly flattened vertebrae

**Fig. 3:**
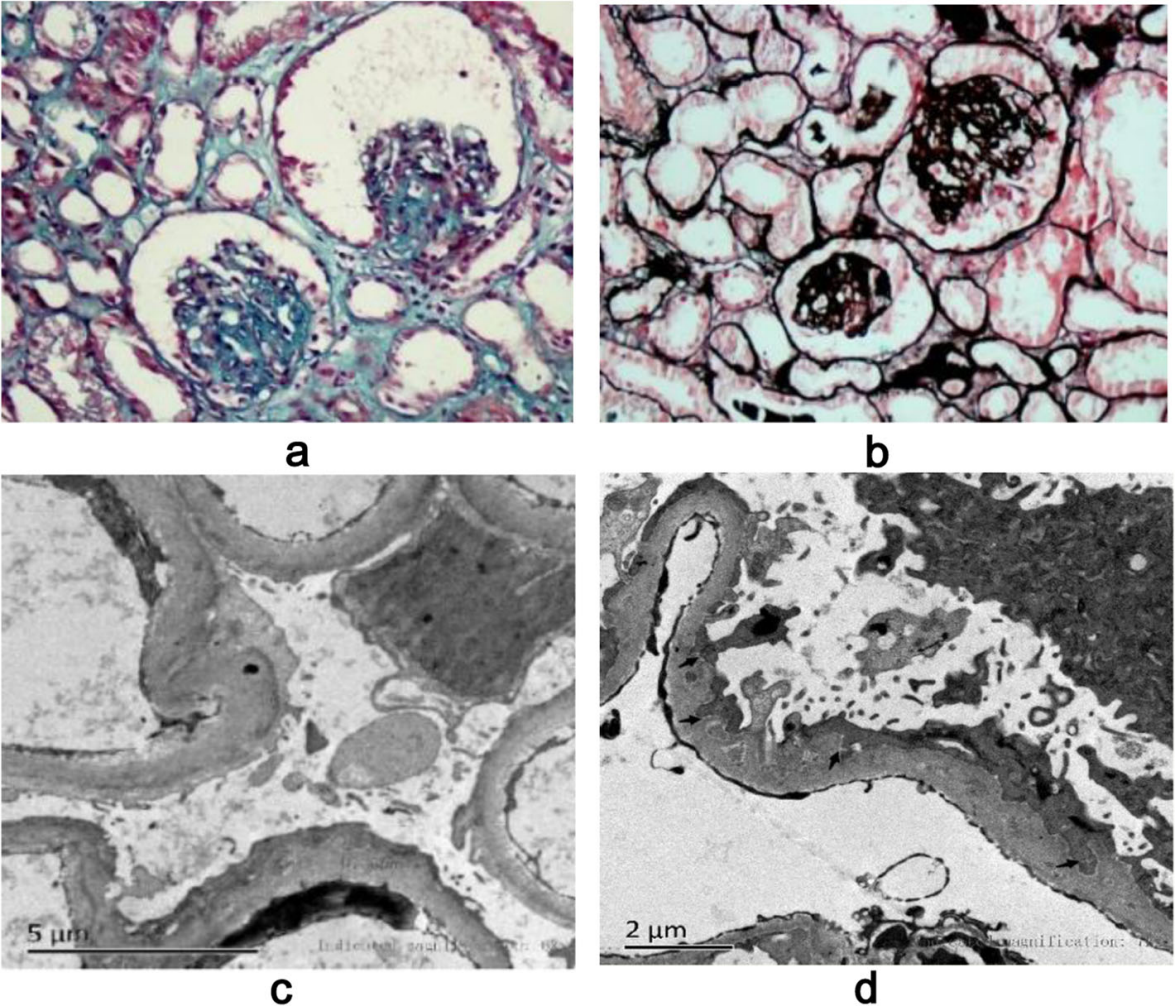
LM shows a glomerulus with segmental sclerosis (**a** and **b**). EM reveals a focal thickness of the GBM without electron-dense deposits. The foot process of podocyte effacement is extensive, with some cytoplasmic processes infolding into the GBM (arrows) (**c** and **d**)

**Fig. 4:**
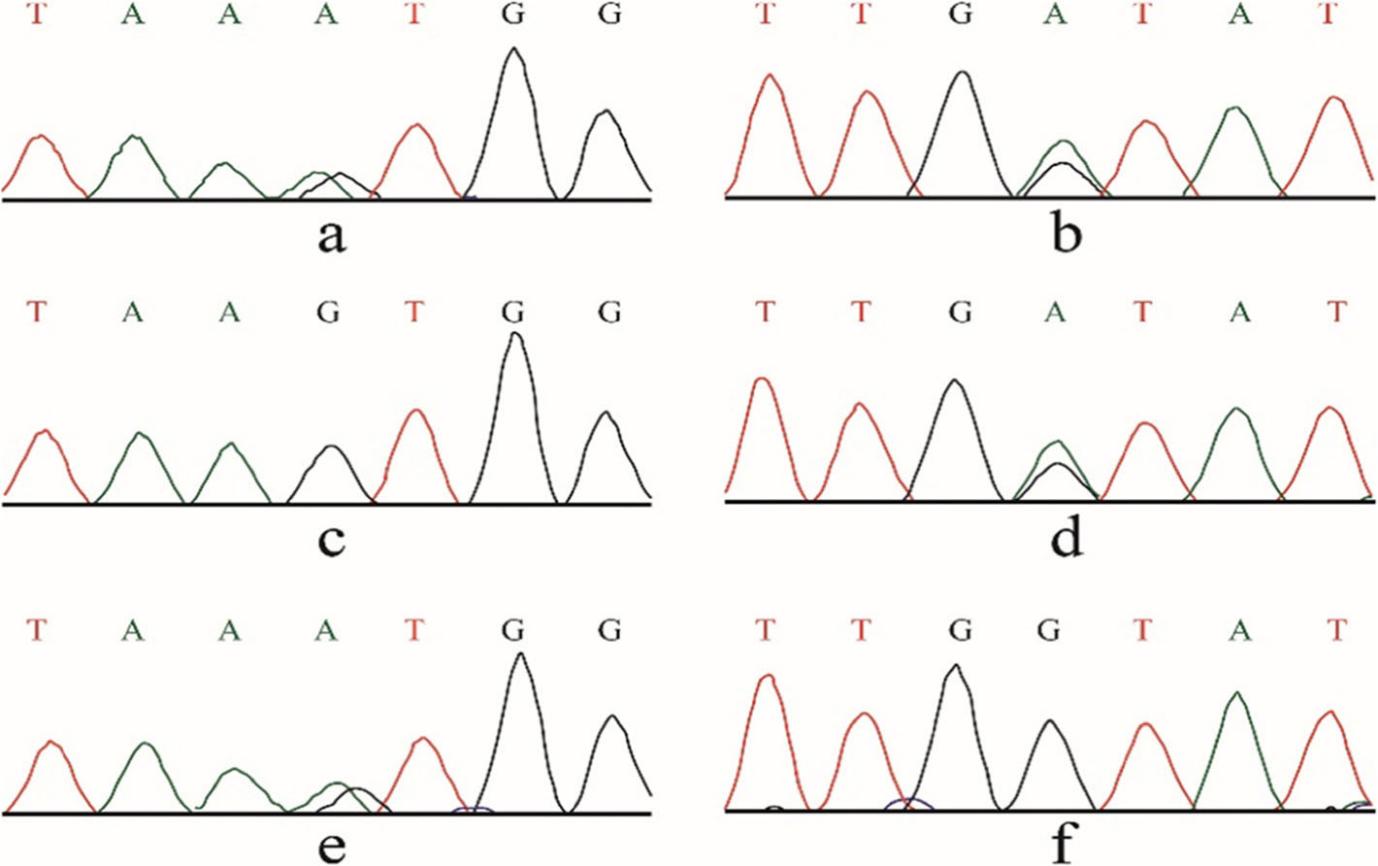
Genetic analysis of the family. Mutation analysis: the patient carries two mutations of the SMARCAL1 gene (**a** and **b**), his mother carries the c.2528 + 1G > A mutation (**c** and **d**), his father carries the c.2141 + 5G > A mutation (**e** and **f**)

The 4-year-old boy was the third child of nonconsanguineous parents and was admitted to our ward in February 2019 for proteinuria and edema lasting 1 month. Both his parents and two older sisters were healthy and had normal stature, and his two brothers were stillborn of unknown cause. He was born at 34 weeks of gestation with a 1-kg birth weight and presented growth retardation. He had a short trunk with a height of 81 cm and a weight of 9.5 kg. The boy demonstrated subtle dysmorphology, with a triangular shape, a broad nasal bridge and a bulbous nasal tip. He had swollen eyelids, lumbar lordosis and a protruding abdomen (Fig. 1). The shifting dullness was negative, and his bilateral lower limbs were swollen. In our department, the laboratory findings were as follows: lymphocytes, 0.5 × 10^9^/L; urine protein, 3.67 g/d (0–0.15 g/d); urine protein/creatinine, 20.1 g/g (0–0.2 g/g); albumin, 9.8 g/L (40.0 g/L-55.0 g/L); cholesterol, 11.72 mmol/L (2.9 mmol/L-5.20 mmol/L); FT3, 0.73 pg/ml (2.00 pg/ml − 4.40 pg/ml); FT4, 0.58 ng/dl (0.93 ng/dl-1.70 ng/dl); and TSH, 10.85 μIU/ml (0.27 μIU/ml-4.20 μIU/ml). The flow cytometry results were as follows: CD3+, 137/μL; CD3 + CD4+, 79/μL; CD3 + CD8+, 7/μL; CD4+/CD8+, 1.54; CD3-CD19+, 405/μL; and CD3-CD16/CD56+, 176/μL. He had no hepatitis infection, and the markers of autoimmunity (ANA, ANCA, dsDNA) were negative. Skeletal X rays showed small iliac wings, small ossification centers of the capital femoral epiphyses, shallow dysplastic acetabular fossae and mildly flattened vertebrae (Fig. 2). He was diagnosed with nephrotic syndrome and hypothyroidism, received 6 weeks of prednisone (17.5 mg/d) and pulse steroid therapy with 100 mg methyl prednisolone for 3 days, and was then started on a combined therapy of steroids and tacrolimus. However, his proteinuria did not improve. During hospitalization, he had influenza A, severe bacterial pneumonia and fungal infection. Because of his special phenotype and resistance to multiple immunosuppressants, a kidney biopsy and gene sequencing were performed. The specimen for LM included twenty-one glomeruli, seven of which exhibited global or focal sclerosis, and some glomeruli were poorly developed (Fig. 3). The deposition of IgA, IgG, IgM, C1q, C3, and C4 by immunofluorescent study (IF) was negative. EM revealed a focal thickness of the GBM (500–2000 nm in thickness) without electron-dense deposits. The foot process of podocyte effacement was extensive, with some cytoplasmic processes infolding into the GBM (Fig. 3). Whole exome sequencing showed novel compound heterozygous mutations in the SMARCAL1 gene (NM_001127207), [5]. Two mutations(c.2141 + 5G > A; c.2528 + 1G > A) were inherited from his parents (Fig. 4). The c.2141 + 5G > A mutation was confirmed to create a novel splice donor site [6]. The c.2528 + 1G > A mutation was not observed in the gnomAD database. According to the ACMG guidelines [7], the c.2528 + 1G > A mutation was classified as likely pathogenic. According to the clinical manifestations and pedigree analysis, we established the diagnosis of SIOD. Given the resistance to steroids and tacrolimus, we stopped these drugs gradually and only treated him with diuretics and angiotensin-converting enzyme inhibitors (ACEIs). After treatment, his edema disappeared gradually, and he was discharged 2 months later. We followed up until June 2019, and the boy did not have edema again and had normal renal function.

## Discussion and conclusions

To date, the pathogenic mechanism of PIG has not been clarified. As almost all the patients with PIG were from East Asia, ethnic or genetic factors might play a role [4]. PIG occurred in most of the patients with underlying immune disorders. Moreover, renal function and proteinuria could be improved by immunosuppressive therapy, supporting the hypothesis of immune abnormalities [8]. Since C5b-9 formation was found by immunoelectron microscopy, some researchers proposed that special types of complements might activate podocytes to infold their process into the GBM, but no autoimmune disorders or abnormal levels of complements were detected in many patients by routine laboratory examinations, and not all biopsies of patients revealed complementary immunoglobulins on LM, indicating that other factors might be involved in the mechanism [9, 10]. GBM materials such as type IV collagen, laminin and heparin sulfate are made mainly by podocytes, and the imbalance between synthesizing and degrading these materials may lead to dysfunction of the GBM, which may trap the foot process more easily [11, 12]. PIG was also found in our patient with mutations in the SMARCAL1 gene. Within the glomerulus, SMARCAL1 localizes to podocytes and endothelial cells, which play a role in the maintenance and integrity of these cells [13]. Further studies are required to understand whether gene mutations, especially those related to podocytes, contribute to the pathogenesis of podocytic infolding.

## Data Availability

The datasets used and/or analysed during the current study are available.

## Abbreviations

SIOD: Schimke immuno-osseous dysplasia
PIG: Podocytic infolding glomerulopathy
GBM: Glomerular basement membrane
EM: Electron microscopy
LM: Light microscopy
FSGS: Focal segmental glomerulosclerosis
ACEIs: Angiotensin-converting enzyme inhibitors
